# Income-related inequality in health insurance coverage: analysis of China Health and Nutrition Survey of 2006 and 2009

**DOI:** 10.1186/1475-9276-11-42

**Published:** 2012-08-14

**Authors:** Jinan Liu, Lizheng Shi, Qingyue Meng, M Mahmud Khan

**Affiliations:** 1 HealthCore, Inc, Wilmington, DE, 19801, USA; 2 Tulane University, School of Public Health and Tropical Medicine, New Orleans, LA, 70112, USA; 3 China Center for Health Development Studies, Peking University, Beijing, 100191, China; 4 Arnold School of Public Health, University of South Carolina, Columbia, SC, 29208, USA

**Keywords:** Inequality, Basic health insurance scheme, Urban resident basic medical insurance, New cooperative medical scheme

## Abstract

**Introduction:**

China introduced the urban resident basic medical insurance (URBMI) in 2007 to cover children and urban unemployed adults, in addition to the new cooperative medical scheme (NCMS) for rural residents in 2003 and the basic health insurance scheme (BHIS) for urban employees in 1998. This study examined whether the overall income-related inequality in health insurance coverage improved during 2006 and 2009 in China.

**Methods:**

The China Health and Nutrition Survey (CHNS) data of 2006 and 2009 were used to create the concentration curve and the concentration index. GEE logistic regression was used to model the health insurance coverage as dependent variable and household income per capita as independent variable, controlling for individuals' age, gender, marital status, educational attainment, employment status, year 2009 (Y2009), household size, retirement status, and geographic variations. The change in the income-related inequality in 2009 was estimated using the interaction term of income*Y2009.

**Results:**

In 2006, 49.7% (4,712/9,476) respondents had health insurance: 13.4% with BHIS and 28.4% with NCMS. In 2009, 90.8% (8,964/9,863) had health insurance: 10.1% with URBMI, 18.3% with BHIS, and 57.6% with NCMS. The BHIS, URBMI, and NCMS programs had different patterns of population coverage over 10 income deciles. The concentration index was 0.15 in 2006 and 0.04 in 2009. The dominance test showed that the concentration curves were significantly different between 2006 and 2009 (p < 0.05). An income increase per capita by 10,000 RMB was associated with 25.5% more likely to have health insurance coverage (odds ratio = 1.255, 95% confidence interval: [1.130-1.393]). In 2009, there was significant improvement in the income-related inequality (p < 0.001).

**Discussions:**

Comparing 2009 to 2006, the income inequality in health insurance coverage was largely corrected in China through rapid expansion of CHNS in rural areas and initiation of URBMI in urban areas.

## Introduction

Despite very rapid growth of the economy during the last decade of the 20^th^ century, the health sector of China remained relatively disorganized and health insurance coverage of the population declined over the years [1]. According to two National Health Services surveys, the percentage of urban and rural populations with health insurance in China decreased to 12% and 9% in 1998 from 53% and 42% in 1993, respectively [2].

To improve health insurance coverage in China, the urban employee Basic Health Insurance Scheme (BHIS) was introduced in urban China in December of 1998. This program, which provides coverage to the employees and retirees in the public sector, remains limited in population coverage [3,4]. The BHIS premium is paid jointly by the employer (six per cent of total wages) and the employee (two per cent of their wage/salary). Local governments are responsible for the management of the BHIS. Employees of the informal sector, unemployed residents, and dependents of employees in urban areas were not eligible for the BHIS. As a result, health insurance coverage remained low in both rural and urban areas even in 2003. By 2003, only 22% of people in urban areas and 13% in rural areas had some forms of health insurance [5].

To improve access to health insurance for rural population, the New Cooperative Medical Scheme (NCMS) was introduced in 2003 [6]. It is operated and organized at the county level. Enrollment in the NCMS is usually based on households rather than individuals enrolling in the program [7]. In 2006, the minimum contributions by governments and households were 40 RMB and 10 RMB per person per year, respectively. The government of China contributed on behalf of poor households to the household component of the premium to ensure equitable financing of the system. In 2009, the average premium was 113 RMB (80% paid by government and 20% paid by rural residents) [8]. In 2011, governmental subsidies increased to 200 RMB per person, and more than 96% population coverage rate (i.e., more than 830 million NCMS members) was reached [5].

The third population group, consisting of 420 million urban residents without formal employment (e.g., students, young children, and unemployed urban residents), were therefore completely left out of the social health insurance until the Urban Resident Basic Medical Insurance (URBMI) was made available in 2007. Enrollment in the URBMI is also on a voluntary basis at the household level. The URBMI premiums are generally higher than those of the NCMS, but lower than those of the BHIS. Government contributions vary depending on the region’s economic status and each individual’s economic situation. Local health insurance bureaus are responsible for determining financing levels. The program was extended from 72 cities in 2007, to 300 cities in 2008 and to all cities of China in 2009. The mean premium of URBMI in 2007 was 236 RMB, 36% of which was paid by the government [9].

In addition to the three main health insurance programs (BHIS, NCMS, and URBMI), a medical financial assistance (MFA) program was also implemented in order to give poor rural and urban households direct support for receiving medical services [10]. Jointly financed by central and local governments, MFA is a highly decentralized program with marked variations across localities.

Despite significant governmental subsidy for health insurance programs such as NCMS and URBMI, the inequalities in health insurance coverage remains a significant concern [11]. Before the introduction of major subsidized programs, income has been positively related to health insurance inequality in China [12]. Although few studies have specifically examined the income-related inequality of health insurance coverage [4,12-14], none has analyzed the income effects of introduction or expansion of insurance programs, especially after URBMI became available. It is important to figure it out if each individual program has been successful in reducing its income dependence. Since all these three programs receive significant subsidy from China government, it is a matter of fairness.

The purpose of this paper was to explore changes in income-based inequality of health insurance coverage in the recent period from 2006 to 2009. We hypothesized that with the introduction or expansion of insurance programs, income has become less important in explaining health insurance coverage than it was in the recent past. To examine the changes in income-related inequality of health insurance coverage by insurance types, this paper also analyzed the three major health insurance programs separately.

## Methods

### Study design

The China Health and Nutrition Survey (CHNS) is an ongoing international collaborative project between the University of North Carolina, Chapel Hill and the China National Institute of Nutrition and Food Safety (http://www.cpc.unc.edu/china). Survey protocols, instruments, and the process for obtaining informed consent for CHNS were approved by the institutional review committees of the University of North Carolina at Chapel Hill and the National Institute of Nutrition and Food Safety, China Center for Disease Control and Prevention. All participants gave their written informed consent. This paper is a secondary data analysis using a public and de-identified dataset, so we did not obtain ethics approval.

The CHNS employed a multistage, random cluster process to draw a sample from nine provinces. A total of two cities and four counties were drawn from each of provinces. The longitudinal CHNS dataset of 2006 and 2009 for this study had information on 19,339 individuals (9,476 in 2006 and 9,863 in 2009). In this study, unbalanced panel data was used in which more than 70% (6,923) participated in both waves.

All participating individuals were asked to complete a structured questionnaire covering: (1) socio-demographics, including age, gender, marital status, education attainment, employment status, occupations, retirement status, and geographic location; (2) annual household income per capita, household size; (3) health insurance coverage type including NCMS, BHIS, and URBMI (since URBMI was a new program, information on it was available only in the 2009 survey).

Annual household income per capita measured in 10,000 RMB was adjusted using consumer price indices between 2006 and 2009. A number of dummy variables were also created for regression analysis: insurance coverage (yes or no), year 2009 (Y2009), gender, marital status (married or not), geographic location (dummy variables for 18 cities and 36 counties), educational attainment (high education: associate/bachelor degree and above; median education: high school; low education: junior high school degree or lower), retired (yes or no), and occupation (farmer, government official (including managerial executive), professional, service sector worker, technician, other occupation, and not working).

### Data analysis

Descriptive statistics (t-tests and Chi-square tests) were calculated for various characteristics of population by insurance coverage. The sample was subdivided into 10 deciles based on household income per capita for 2006 and 2009 separately to understand the health insurance coverage by income deciles and its change over the years 2006 to 2009.

#### Concentration curve and concentration index

Concentration curves and concentration indices [15] for both 2006 and 2009 were used to summarize the degree of income-related inequality by type of health insurance coverage. A concentration curve plots the cumulative percentage of health insurance (y-axis) against the cumulative percentage of population ranked by income deciles, from the poorest to the richest (x-axis). If health insurance coverage takes higher values among richer people, the concentration curve is below the line of equality (the 45-degree line). The farther the curve is below the line of equality, the higher is the income-related inequality in favor of richer segments of the population (pro-rich distribution). In this case, the concentration index is positive. If the outcome proportions are higher for the poorer groups, the concentration index is negative. In addition, a dominance test was used to see if concentration curves for 2006 and 2009 were significantly different from each other.

#### Modeling

Generalized estimating equation (GEE) regression for repeated measure data [16] in difference-in-difference format [3] was used for the analysis. The model was specified as:

(1) $$\begin{array}{l} y=\beta_{0}+\beta_{1}*\text{Income}+\beta_{2}*Y2009\\ \;\;+\delta_{1}\left(\text{Income}*Y2009\right)+\beta_{i}X_{i}+u \end{array}$$

where *y* was a dichotomous health insurance coverage variable, *Y2009* was the dummy for 2009. The dummy *Y2009 (β*_*2*_*)* captured temporal factors that would cause changes in *y* in 2009 including the effects of URBMI as a new insurance program and other structural changes. Meanwhile, *β*_*1*_ captured the overall effect of income on insurance coverage in 2006; *(β*_*1 +*_*δ*_*1*_*)* captured the overall effect of income on insurance coverage in 2009; therefore, *δ*_*1*_ captured the change in income-related inequality in insurance coverage from 2006 to 2009. *X*_*i*_ included individuals’ age, gender, marital status, educational attainment, retirement status, occupation, household size, and geographic dummies (18 cities and 36 counties). Self-correlation between 6,923 individuals included in both waves was also controlled for in the GEE model. A sensitivity analysis was performed by using hierarchical logistic regression [17], which controlled for variations among different counties and provinces in terms of insurance policy, economic development, etc.

## Results

In 2006, 49.7% (4,712/9,476) participants had health insurance including 13.4% with BHIS and 28.4% with NCMS. The population health insurance coverage was 90.8% (8,964/9,863) in 2009. Specifically, in 2009, 10.1% individuals reported having URBMI coverage, 18.3% had BHIS, and 57.6% had NCMS. In 2009, at least one person carried URBMI for each of all the cities and counties in this survey. This result suggested that all the cities in the survey had URBMI program in place by 2009. We also found 4.8% of total respondents in 2009 had “other” insurance programs including 2.8% in commercial insurance market and the remaining covered by special programs (e.g., maternal child health, vaccination, etc.). However, the MFA program was not specified in the CHNS survey.

Tables 1 and 2 present the descriptive information for 2006 and 2009, comparing the characteristics of insured (by type) and uninsured groups. In 2006 and 2009, people with insurance were consistently older, with higher household income per capita, more likely to be married, and better educated. The differences in the characteristics of beneficiaries across three insurance programs were also evident by area of residence, educational status, occupation, and income.

**Table 1:** Descriptive information between uninsured and insured groups in 2006 and 2009

	**Year 2006**		**P values in 2006**	**Year 2009**		**P values in 2009**
	**(N = 9476)**			**(N = 9863)**		
	**Uninsured**	**Insured**		**Uninsured**	**Insured**	
Number of observations	4764	4712		899	8964	
Residence, n (%)			0.006			<0.0001
Urban	1549 (32.51)	1657 (35.17)		442 (49.17)	2878 (32.11)	
Rural	3215 (67.49)	3055 (64.83)		457 (50.83)	6086 (67.89)	
Gender, n (%)			0.001			0.191
Male	2188 (45.93)	2323 (49.30)		413 (45.94)	4323 (48.23)	
Female	2576 (54.07)	2389 (50.70)		486 (54.06)	4641 (51.77)	
Marital status, n (%)			<0.0001			<0.0001
Unmarried	916 (19.23)	643 (13.65)		248 (27.59)	1388 (15.48)	
Married	3848 (80.77)	4069 (86.35)		651 (72.41)	7576 (84.52)	
Education attainment, n (%)			<0.0001			<0.0001
Low education	3769 (79.11)	3183 (67.55)		627 (69.74)	6778 (75.61)	
Median education	864 (18.14)	1126 (23.90)		218 (24.25)	1697 (18.93)	
High education	131 (2.75)	403 (8.55)		54 (6.01)	489 (5.46)	
Retirement, n (%)			<0.0001			<0.0001
Not retired	4470 (93.83)	3849 (81.69)		822 (91.43)	7786 (86.86)	
Retired	294 (6.17)	863 (18.31)		77 (8.57)	1178 (13.14)	
Occupations, n (%)			<0.0001			<0.0001
Officials	119 (2.50)	370 (7.85)		31 (3.45)	473 (5.28)	
Professionals	94 (1.97)	347 (7.36)		24 (2.67)	456 (5.09)	
Technicians	337 (7.07)	537 (11.40)		80 (8.90)	859 (9.58)	
Farmers	1434 (30.10)	1218 (25.85)		88 (9.79)	2590 (28.89)	
Service sector workers	510 (10.71)	328 (6.96)		151 (16.80)	777 (8.67)	
Others	169 (3.55)	130 (2.76)		43 (4.78)	262 (2.92)	
Not working	2101 (44.10)	1782 (37.82)		482 (53.62)	3547 (39.57)	
Age in years, mean (SD)	48.5 (15.9)	50.3 (14.6)	<0.0001	47.7 (17.9)	50.4 (15.3)	<0.0001
Household size, mean (SD)	3.9 (1.69)	3.5 (1.48)	<0.0001	3.8 (1.69)	3.7 (1.65)	0.053
Household income per capita in 10000 RMB, mean (SD)	0.62 (0.91)	1.00 (1.24)	<0.0001	0.89 (1.01)	1.18 (1.54)	<0.0001

**Table 2:** Descriptive information of population with different types of insurance in 2006 and 2009

	**Year 2006**		**Year 2009**		
	**NCMS**^**a**^	**BHIS**^**b**^	**NCMS**	**BHIS**	**URBMI**^**c**^
Number of observations	2694	1274	5683	1807	999
Residence, n (%)					
Urban	305 (11.32)	898 (70.49)	919 (16.17)	1196 (66.19)	496 (49.65)
Rural	2389 (88.68)	376 (29.51)	4764 (83.83)	611 (33.81)	503 (50.35)
Gender, n (%)					
Male	1246 (46.25)	660 (51.81)	2658 (46.77)	972 (53.79)	415 (41.54)
Female	1448 (53.75)	614 (48.19)	3025 (53.23)	835 (46.21)	584 (58.46)
Marital status, n (%)					
Unmarried	368 (13.66)	157 (12.32)	848 (14.92)	247 (13.67)	200 (20.02)
Married	2326 (86.34)	1117 (87.68)	4835 (85.08)	1560 (86.33)	799 (79.98)
Education attainment, n (%)					
Low education	2333 (86.60)	503 (39.48)	5109 (89.90)	796 (44.05)	691 (69.17)
Median education	340 (12.62)	504 (39.56)	547 (9.63)	712 (39.40)	260 (26.03)
High education	21 (0.78)	267 (20.96)	27 (0.48)	299 (16.55)	48 (4.80)
Retirement, n (%)					
Not retired	2649 (98.33)	748 (58.71)	5612 (98.75)	1056 (58.44)	806 (80.68)
Retired	45 (1.67)	526 (41.29)	71 (1.25)	751 (41.56)	193 (19.32)
Occupations, n (%)					
Officials	46 (1.71)	210 (16.48)	95 (1.67)	257 (14.22)	49 (4.90)
Professionals	40 (1.48)	228 (17.90)	67 (1.18)	287 (15.88)	22 (2.20)
Technicians	309 (11.47)	148 (11.62)	517 (9.10)	214 (11.84)	69 (6.91)
Farmers	1188 (44.10)	3 (0.24)	2545 (44.78)	3 (0.17)	39 (3.90)
Service sector workers	196 (7.28)	69 (5.42)	446 (7.85)	134 (7.42)	169 (16.92)
Others	88 (3.27)	27 (2.12)	155 (2.73)	52 (2.88)	33 (3.30)
Not working	827 (30.70)	589 (46.23)	1858 (32.69)	860 (47.59)	618 (61.86)
Age in years, mean (SD)	48.9 (14.2)	52.4 (14.2)	49.3 (15.1)	52.5 (14.6)	52.4 (15.7)
Household size, mean (SD)	3.8 (1.5)	3.1 (1.3)	4.0 (1.7)	3.1 (1.3)	3.4 (1.5)
Household income per capita in 10000 RMB, mean (SD)	0.72 (1.21)	1.42 (1.09)	0.93 (1.35)	1.74 (1.56)	1.17 (1.16)

^a^ Basic health insurance scheme (employee).

^b^ New cooperative medical scheme.

^c^ Urban resident basic medical insurance.

For these 6923 individuals who participated in both waves, the health insurance coverage rate increased from 50.92% in 2006 to 92.40% in 2009. Also the household income per capita increased from 8100 RMB in 2006 to 11600 RMB in 2009. A little bit more people with high education (4.52% vs. 4.32%) and less people working (58.40% vs. 60.80%) and more people retired (13.29% vs. 11.31%) in 2009. No changes in residence or household size have been observed.

**Figure 1:**
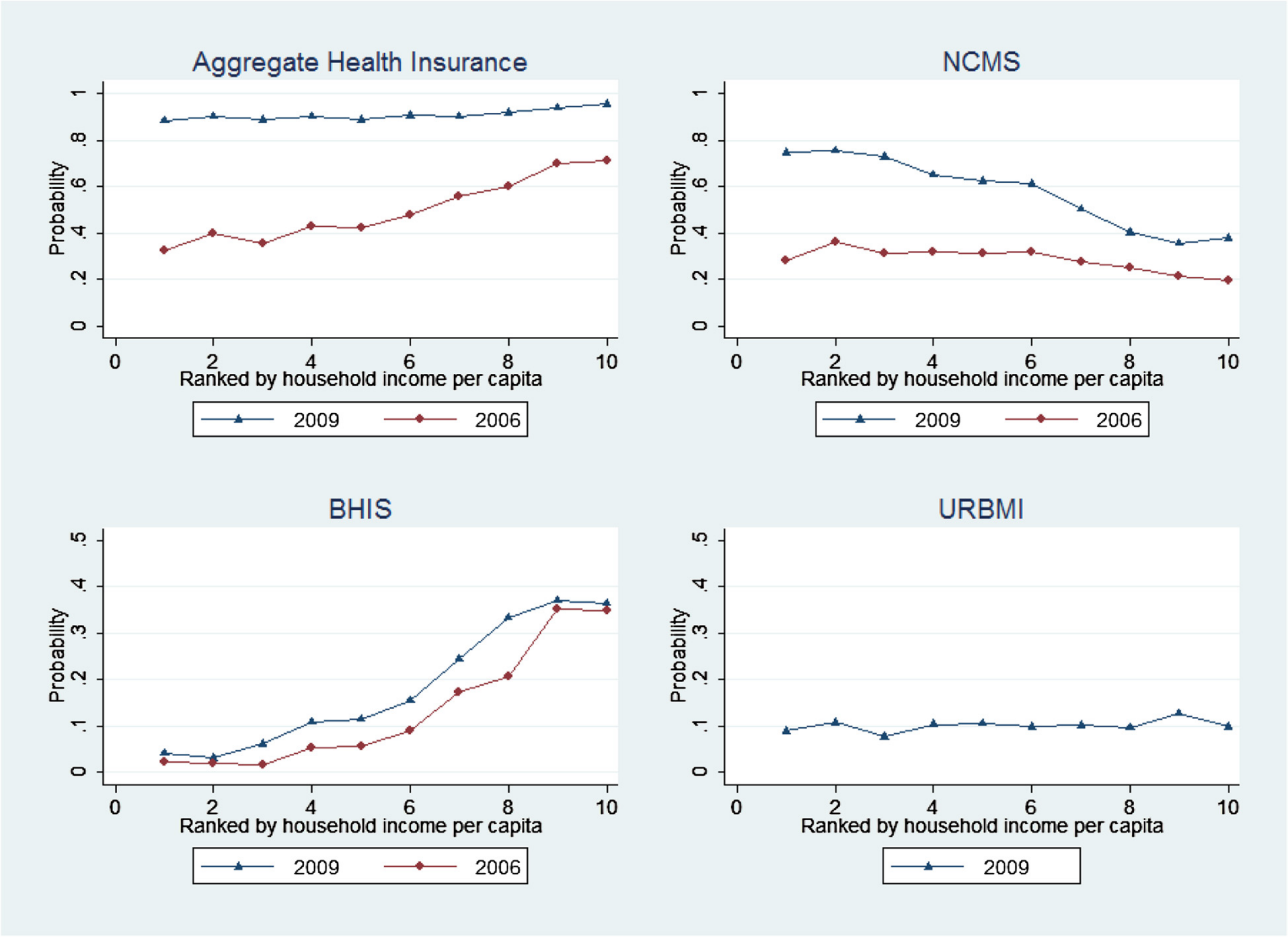
Insurance coverage rate in 2006 and in 2009 in 10 deciles ranked by annual household income per capita.

Figure 1 displays the health insurance coverage rates by per-capita income deciles from the poorest (one) to the richest (ten). In 2006, the health insurance coverage rate was significantly higher for the higher income groups compared to the low-income groups. Across income deciles, insurance coverage rates ranged from 32.2% for the poorest docile to 70.0% for the richest docile. Interestingly, the pattern of health insurance coverage by income deciles was reverse for NCMS, i.e., the coverage was higher for the poorest income groups compared to other higher income groups. Therefore, the overall pro-rich pattern of income-related inequality of insurance coverage in 2006 was driven by the BHIS and if the NCMS program had not been there, the coverage inequality would have been significantly higher. However, the aggregate health insurance coverage line became quite flat in 2009 (ranging from 88.5% for the first docile to 95.5% for the tenth docile). The URBMI coverage line was also more or less flat around 10% across all income groups. The BHIS in 2009 maintained an increasing trend in coverage with respect to income groups as it was in 2006. Meanwhile, the NCMS coverage rate was downward sloping, indicating that the scheme has been pro-poor in nature (insurance coverage declining from 74.6% for the lowest income docile to 37.9% for the top docile).

**Figure 2:**
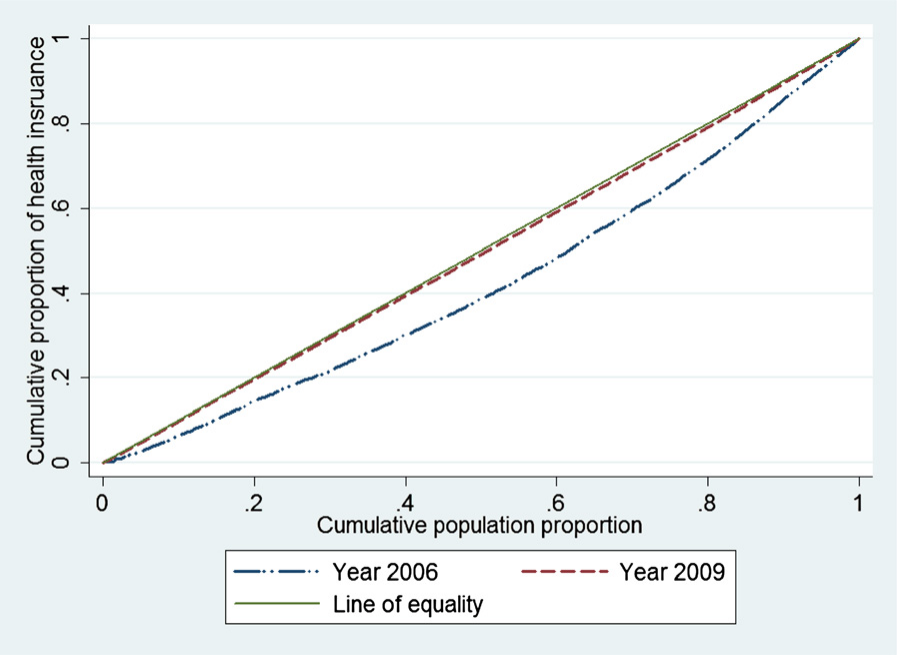
Concentration curves for health insurance coverage in China, 2006 and 2009.

Figure 2 presents the concentration curves for health insurance coverage in 2006 and 2009. Both the curves were below the line of equality indicating that the health insurance coverage has remained pro-rich in both years, although significant reduction in income-related inequality happened over a short span of three years. The dominance test showed that the two curves were significantly apart from each other (p < 0.05). The concentration indices were 0.15 in 2006 and 0.04 in 2009. Note that the income-related inequality was very close to zero in 2009, mainly because of high degree of health insurance coverage achieved by 2009.

Table 3 presents the difference-in-difference results from both the GEE logistic model and hierarchical logistic model. In 2009, respondents were almost 23 folds more likely to have health insurance compared to 2006, reflecting the enormous impact of health insurance reforms had on insurance coverage, possibly through the introduction of URBMI and other structural changes since 2006. Controlling for the effects of geographic locations (18 cities and 36 counties) and individuals’ demographics, household income per capita was a significant predictor of health insurance coverage. Higher household income per capita by 10,000 RMB increased the probability of having insurance coverage by more than 25.5% in 2006 (odds ratio (OR) =1.255, 95% confidence interval (CI): 1.130-1.393). The interaction term indicated that the effect of income reduced significantly, by 20.3% in 2009 for the same increase in income (OR: 0.797, 95% CI: 0.714-0.890, p < 0.001). In other words, positive effect of income on insurance coverage rate observed in 2006 was completely erased in 2009 (OR = 1.000, 95% CI: 0.956-1.046). The hierarchical model showed consistent results as the GEE logistic model.

**Table 3:** Predictors of health insurance coverage according to GEE model and hierarchical logistic regression in difference-in-difference format

	**GEE model**				**Hierarchical model**			
	**Odds Ratio**	**Lower 95% CI**	**Upper 95% CI**	**P value**	**Odds Ratio**	**Lower 95% CI**	**Upper 95% CI**	**P value**
Intercept	5.288	3.074	9.097	<0.001	4.924	3.021	8.026	<0.001
Age	1.008	1.005	1.011	<0.001	1.008	1.005	1.011	<0.001
Household size	0.998	0.971	1.026	0.908	0.997	0.969	1.026	0.894
Male	1.000	0.921	1.086	0.996	0.999	0.918	1.088	0.981
Not married	0.740	0.660	0.829	<0.001	0.739	0.661	0.826	<0.001
Low education	0.717	0.562	0.915	0.007	0.719	0.572	0.904	0.005
Median Education	0.692	0.545	0.879	0.003	0.694	0.555	0.87	0.002
Year of 2009	22.809	20.041	25.959	<0.001	17.903	16.175	19.816	<0.001
Income increase by 10 K RMB	1.255	1.130	1.393	<0.001	1.255	1.177	1.339	<0.001
Interaction of income*Y2009	0.797	0.714	0.890	<0.001	0.797	0.735	0.865	<0.001
Not retired	0.216	0.178	0.263	<0.001	0.219	0.185	0.259	<0.001
Occupation (Technicians as reference)								
Professionals	2.413	1.766	3.297	<0.001	2.392	1.807	3.167	<0.001
Officials	2.187	1.656	2.887	<0.001	2.166	1.673	2.804	<0.001
Farmers	0.892	0.752	1.058	0.190	0.889	0.752	1.052	0.170
Service sector workers	0.464	0.378	0.570	<0.001	0.464	0.384	0.561	<0.001
Others	0.555	0.424	0.728	<0.001	0.555	0.427	0.722	<0.001
Not working	0.423	0.354	0.505	<0.001	0.423	0.358	0.501	<0.001
Set of 53 geographic locations	^a^	^a^	^a^	^a^	^b^	^b^	^b^	^b^
(City 1 in Liaoning as reference)								

^a^ The GEE estimates for 53 variables for geographic locations are omitted; the full GEE model is presented in the Appendix.

^b^ Random effect of geographic variations (18 cities and 36 counties) was controlled for hierarchical model.

## Discussions

Our income-related inequality study on China’s health reforms enriches the general literature of social health insurance [18,19]. The income-related inequality in health insurance coverage rate has been significantly reduced in 2009 through the expansion of CHNS in rural areas and introduction of URBMI in urban areas. By 2009, China has achieved close to universal coverage (i.e., almost 91% of total population in the CHNS survey) by adopting complementary insurance programs to target different groups of individuals in the country. Each program has its own specific target groups and the reforms were designed to provide different levels of incentives to participate in the insurance programs based on the economic status of the potential participants. Meanwhile, all the three major health insurance programs adopted a decentralized local-level management system based on the general framework suggested at the national level. The central government’s subsidy has created enough incentives for all local governments to participate and the system of differential premium subsidy based on individual income and economic development levels of geographic regions (East, Middle, and West) was also successful in attracting the lower income groups in the insurance programs. Inequality in health insurance coverage is a common challenge worldwide, especially in countries with decentralized insurance system [20,21]. The success in tackling income dependence of health insurance in China provides useful learning materials for other countries still face similar challenges.

People without health insurance in China usually have less access to health service [22] and more out of pocket expense cost [11,23]. Income has been positively related to health insurance disparity in China [12,24,25]. Generally, the economically disadvantaged groups have less health insurance coverage [21]. The disparity in insurance coverage had once been narrowed significantly across socioeconomic groups during 1989-1997 [12]. Even during 1991-2000, health insurance coverage gap related to unbalanced economic development decreased between urban and rural employees [24]. However, a new study reported that high income groups were more likely to carry health insurance during 1998 to 2003 and disparity in health insurance coverage has been not reduced in spite of rapid economic development and the urban health insurance reform efforts during this period [4]. Our empirical study indicates that the aggregate health insurance coverage has become almost independent of household income in 2009, although significant income-related inequality existed in 2006. Consistent with the previous findings [14,23], the concentration curve in 2006 was found to be significantly below the equality line, meaning that the health insurance coverage was pro-rich in 2006. Since the health insurance coverage has reached more than 90% level, it is not surprising that income-related inequality has declined drastically. Actually, income was no longer a significant predictor of health insurance coverage in 2009. In addition to the “pro-poor” mechanisms of the NCMS, it has been reported that the poorer participants have received higher level of subsidies from URBMI and felt more satisfied with it [9]. It indicates that current URBMI works well so far to expand the insurance coverage and further to tackle inequality in health insurance coverage between the rich and the poor. Therefore, the substantial investment in health by Chinese government to achieve universal coverage has been very successful (112 billion RMB in 2003, 240 billion RMB in 2008, and additional 850 billion RMB over three years since 2009) [26]. It will be important, however, to see if insurance coverage can be sustained in the future.

The different patterns in income-related inequality observed for three health insurance types are an interesting finding. This probably indicates how the health reform in China effectively created complementary programs to cover those who were once left out by BHIS and possibly commercial health insurance plans. The NCMS was designed to cover the rural population with higher degree of subsidy provided to the poor members. In fact, since the NCMS has been so successful in recruiting the poorer groups, few case studies on local-level premium subsidy policies should be useful for other developing economies of the world. Another important point to note is that implementation of a potential pro-poor program may not show pro-poor outcomes during the first few years of its implementation. For example, the NCMS coverage rate curve in 2006 does not show higher coverage for the poorer sections of rural areas; in fact, the curve remained flat by income deciles in 2006 (Figure 1). Only after a very significant governmental push for improved coverage of the NCMS as top priority of recent health reforms, the function of coverage rate did become pro-poor by 2009.

Interestingly, there were slightly more than 10% NCMS participants with urban residence. NCMS intends to cover rural areas only, and all participants need to have official status of a rural residence (“nongcun hukou”). Further, its insufficiency of benefit packages and high amount of out-of-pocket costs made it unattractive among rich people. In this public research data, we cannot make a conclusion why these “urban people” enrolled in NCMS. One possible explanation could be these people migrated from rural to urban and lived in urban areas without permanent urban residence.

Uninsured workers in urban areas should be the target population for future health reforms [27]. In this study, the income level may still be a significant predictor of the BHIS coverage even in 2009 as Figure 1 presents the same patterns for both years. It indicates that the current individual financial contribution to BHIS pool still poses heavy burden on the poor workers (such as informal sector workers). The poor workers may be left behind as the uninsured even after three insurance programs were made available. China government may consider new policy options: increasing subsidies to effectively expand the BHIS coverage to urban poor workers or introducing changes in insurance attributes to allow the NCMS portability into the URBMI for migrant workers.

Despite substantially improved equality in insurance coverage at the national level as presented in this study, inequality in other dimensions, such as health care utilization, morbidity and mortality, may not be improved simultaneously. The gains in health outcome and health access under the current health reforms were uneven and limited (e.g., concerns of high out of pocket cost and delaying/forgoing treatment) [28-30]. However, health insurance coverage is a very important and necessary means to achieve the ultimate health outcome. In addition, reducing the gap in health outcomes between rural and urban areas in China has been a focus of the health reform efforts since 2002 [31]. Therefore, future priorities of health reforms could focus on improving health care equalities in outcomes, controlling health care cost [32], and addressing major concerns on the benefit packages of health insurance and payment fairness. The discussion about integrating CHNS and URBMI to tackle the challenges of rural–urban migrate and urbanization in China is very heated[33]. In our opinion, it is important for the policy makers to understand the relationship between income and health insurance rate in each type of plans before moving forward to integrate them. This analysis of all these three major programs concurrently could be very useful.

This is a retrospective observational study with its own limitations. First, although we controlled the geographic variations, there are some unobservable structures and status of the population including local economic development, job market, social protection policies/programs in different areas, and the variation in the BHIS benefits. Second, this study is not a representative sample of China despite use of clustered randomization sampling. Cautions should be excised in generalizing the results to other populations and the entire country. Lastly, the CHNS did not have specified the MFA coverage, while this program was also designed to reduce income dependence of health insurance coverage.

In summary, the overall income-related inequality in health insurance coverage existed in 2006 was almost corrected in 2009 through rapid expansion of CHNS in rural areas and initiation of URBMI in urban areas. Pro-rich nature of BHIS represents an opportunity to further reduce the income dependence and improve the fairness in China health systems. In addition, further studies on health care access and outcomes beyond health insurance coverage are warranted as health reforms evolve.

## Appendix

**Table 4:** Predictors of health insurance coverage according to GEE model and hierarchical logistic regression in difference-in-difference format

	**GEE model**				**Hierarchical model**			
	**Odds Ratio**	**Lower 95% CI**	**Upper 95% CI**	**P value**	**Odds Ratio**	**Lower 95% CI**	**Upper 95% CI**	**P value**
Intercept	5.288	3.074	9.097	<0.001	4.924	3.021	8.026	<0.001
Age	1.008	1.005	1.011	<0.001	1.008	1.005	1.011	<0.001
Household size	0.998	0.971	1.026	0.908	0.997	0.969	1.026	0.894
Male	1.000	0.921	1.086	0.996	0.999	0.918	1.088	0.981
Not married	0.740	0.660	0.829	<0.001	0.739	0.661	0.826	<0.001
Low education	0.717	0.562	0.915	0.007	0.719	0.572	0.904	0.005
Median Education	0.692	0.545	0.879	0.003	0.694	0.555	0.87	0.002
Year of 2009	22.809	20.041	25.959	<0.001	17.903	16.175	19.816	<0.001
Income increase by 10000 RMB	1.255	1.130	1.393	<0.001	1.255	1.177	1.339	<0.001
Interaction of income*Y2009	0.797	0.714	0.890	<0.001	0.797	0.735	0.865	<0.001
Not retired	0.216	0.178	0.263	<0.001	0.219	0.185	0.259	<0.001
Occupation (Technicians as reference)								
Professionals	2.413	1.766	3.297	<0.001	2.392	1.807	3.167	<0.001
Officials	2.187	1.656	2.887	<0.001	2.166	1.673	2.804	<0.001
Farmers	0.892	0.752	1.058	0.190	0.889	0.752	1.052	0.170
Service sector workers	0.464	0.378	0.570	<0.001	0.464	0.384	0.561	<0.001
Others	0.555	0.424	0.728	<0.001	0.555	0.427	0.722	<0.001
Not working	0.423	0.354	0.505	<0.001	0.423	0.358	0.501	<0.001
Geographic locations (City 1 in Liaoning Province as reference)								
City 2 in Liaoning Province	0.674	0.408	1.112	0.122	^a^	^a^	^a^	^a^
County 1 in Liaoning Province	2.000	1.176	3.402	0.011	^a^	^a^	^a^	^a^
County 2 in Liaoning Province	4.886	2.548	9.371	<0.001	^a^	^a^	^a^	^a^
County 3 in Liaoning Province	1.999	1.181	3.382	0.01	^a^	^a^	^a^	^a^
County 4 in Liaoning Province	1.045	0.621	1.757	0.869	^a^	^a^	^a^	^a^
City 1 in Heilongjiang	0.384	0.247	0.597	<0.001	^a^	^a^	^a^	^a^
City2 in Heilongjiang	0.420	0.267	0.661	<0.001	^a^	^a^	^a^	^a^
County 1 in Heilongjiang	2.749	1.420	5.321	0.003	^a^	^a^	^a^	^a^
County 2 in Heilongjiang	0.113	0.071	0.179	<0.001	^a^	^a^	^a^	^a^
County 3 in Heilongjiang	1.916	1.104	3.324	0.021	^a^	^a^	^a^	^a^
County 4 in Heilongjiang	2.587	1.441	4.644	0.001	^a^	^a^	^a^	^a^
City 1 in Jiangsu	4.891	2.620	9.130	<0.001	^a^	^a^	^a^	^a^
City2 in Jiangsu	0.389	0.239	0.634	<0.001	^a^	^a^	^a^	^a^
County 1 in Jiangsu	6.468	3.551	11.784	<0.001	^a^	^a^	^a^	^a^
County 2 in Jiangsu	4.254	2.323	7.791	<0.001	^a^	^a^	^a^	^a^
County 3 in Jiangsu	1.022	0.604	1.729	0.936	^a^	^a^	^a^	^a^
County 4 in Jiangsu	7.741	4.188	14.311	<0.001	^a^	^a^	^a^	^a^
City 1 in Shandong	0.811	0.479	1.373	0.436	^a^	^a^	^a^	^a^
City2 in Shandong	0.401	0.246	0.655	<0.001	^a^	^a^	^a^	^a^
County 1 in Shandong	6.542	3.581	11.954	<0.001	^a^	^a^	^a^	^a^
County 2 in Shandong	0.263	0.174	0.398	<0.001	^a^	^a^	^a^	^a^
County 3 in Shandong	2.793	1.620	4.812	<0.001	^a^	^a^	^a^	^a^
County 4 in Shandong	15.210	7.369	31.394	<0.001	^a^	^a^	^a^	^a^
City 1 in Henan	1.031	0.625	1.700	0.906	^a^	^a^	^a^	^a^
City2 in Henan	0.445	0.281	0.705	0.001	^a^	^a^	^a^	^a^
County 1 in Henan	0.248	0.160	0.383	<0.001	^a^	^a^	^a^	^a^
County 2 in Henan	0.301	0.199	0.457	<0.001	^a^	^a^	^a^	^a^
County 3 in Henan	2.219	1.346	3.657	0.002	^a^	^a^	^a^	^a^
County 4 in Henan	0.242	0.158	0.371	<0.001	^a^	^a^	^a^	^a^
City 1 in Hubei	0.914	0.562	1.486	0.717	^a^	^a^	^a^	^a^
City2 in Hubei	0.493	0.301	0.809	0.005	^a^	^a^	^a^	^a^
County 1 in Hubei	0.153	0.098	0.239	<0.001	^a^	^a^	^a^	^a^
County 2 in Hubei	2.491	1.489	4.169	0.001	^a^	^a^	^a^	^a^
County 3 in Hubei	4.937	2.535	9.616	<0.001	^a^	^a^	^a^	^a^
County 4 in Hubei	15.349	7.374	31.950	<0.001	^a^	^a^	^a^	^a^
City 1 in Hunan	1.123	0.685	1.842	0.645	^a^	^a^	^a^	^a^
City2 in Hunan	0.568	0.350	0.924	0.023	^a^	^a^	^a^	^a^
County 1 in Hunan	0.246	0.160	0.377	<0.001	^a^	^a^	^a^	^a^
County 2 in Hunan	1.409	0.843	2.357	0.191	^a^	^a^	^a^	^a^
County 3 in Hunan	0.228	0.148	0.352	<0.001	^a^	^a^	^a^	^a^
County 4 in Hunan	0.287	0.184	0.449	<0.001	^a^	^a^	^a^	^a^
City 1 in Guangxi	0.186	0.113	0.308	<0.001	^a^	^a^	^a^	^a^
City2 in Guangxi	0.231	0.146	0.367	<0.001	^a^	^a^	^a^	^a^
County 1 in Guangxi	0.887	0.537	1.464	0.638	^a^	^a^	^a^	^a^
County 2 in Guangxi	0.426	0.257	0.707	0.001	^a^	^a^	^a^	^a^
County 3 in Guangxi	0.711	0.434	1.163	0.174	^a^	^a^	^a^	^a^
County 4 in Guangxi	0.325	0.213	0.496	<0.001	^a^	^a^	^a^	^a^
City 1 in Guizhou	1.163	0.712	1.899	0.546	^a^	^a^	^a^	^a^
City2 in Guizhou	0.502	0.295	0.855	0.011	^a^	^a^	^a^	^a^
County 1 in Guizhou	2.719	1.564	4.725	<0.001	^a^	^a^	^a^	^a^
County 2 in Guizhou	0.210	0.136	0.323	<0.001	^a^	^a^	^a^	^a^
County 3 in Guizhou	0.256	0.166	0.393	<0.001	^a^	^a^	^a^	^a^
County 4 in Guizhou	1.071	0.656	1.750	0.784	^a^	^a^	^a^	^a^

^a^ Random effect of geographical locations (18 cities and 36 counties) was controlled for hierarchical model.

## Competing interests

All authors declare no competing interests.

## Authors’ contributions

JL and LS jointly conceptualized the study design, drafted the manuscript and should be considered as joint first authors. JL performed all data analyses. MK provided guidance and advice on methodology for data analysis and provided detailed comments on the manuscript. QM provided help in interpreting the data and comments on the manuscript. All authors read and approved the final manuscript.
